# The future of European outdoor summer sports through the lens of 50 years of the Tour de France

**DOI:** 10.1038/s41598-025-30129-8

**Published:** 2026-02-24

**Authors:** Ivana Cvijanovic, James D. Begg, Malcolm N. Mistry, Desislava Petrova, Chloe Brimicombe, Benjamin Sultan

**Affiliations:** 1 ESPACE-DEV, Univ Montpellier IRD Univ Guyane Univ Reunion Univ Antilles Univ Avignon, 500 rue Jean-François Breton, 34093 Montpellier, France; 2 Galson Sciences Limited, Oakham, 9 Melton Rd, LE16 6AX UK; 3 Environment & Health Modelling (EHM) Department of Public Health Environments and Society, London School of Hygiene & Tropical Medicine, London, 15-17 Tavistock Place, WC1H 9SH UK; 4 Department of Economics, Ca’ Foscari University of Venice, Venice, Cannaregio 873/b 30121 Italy; 5 Climate and Health Group (CANU), Barcelona Institute for Global Health (ISGlobal), Barcelona, Doctor Aiguader 88, 08003 Spain; 6 Royal Meteorological Society, Reading, 108 Oxford Road, RG1 7LL UK

**Keywords:** Climate sciences, Climate change, Climate-change impacts, Environmental health

## Abstract

In this study, we consider how excessive heat, due to changing climate conditions, could affect the safety of summer sport competitions in Europe. We use the example of the world’s largest bicycle race, the Tour de France, to assess the changes in heat stress over the past five decades and discuss extreme heat protocols, data gaps and safe ways forward. We show that although July heat stress values have been increasing throughout France, the actual Tour de France race dates have so far been fortunate to avoid the days featuring the highest heat. For example, although July hourly heat stress values for Paris and its surroundings have crossed the high-risk threshold (Wet Bulb Globe Temperature (WBGT) > 28 °C) on five occasions over the last 50 years, four of which have occurred since 2014, this has so far not happened on the date of the Tour de France stage through Paris. Between 1974 and 2023, the hottest Parisian stage finish was in July 2002, with the hourly WBGT maximum of 26.8 °C staying just below the high-risk mark. With record-breaking heatwaves becoming more frequent, it seems only a question of time as to when the race will encounter the extreme heat stress days that will test the existing heat safety protocols. Considering the historical heat stress values, we find that the episodes of dangerous heat levels exceeding 28 °C WBGT, have been most common around Toulouse, Pau and Bordeaux in France’s southwest, and around Nîmes and Perpignan in the southeast. However, locations like Paris and Lyon are starting to cross the 28 °C WBGT threshold more frequently, becoming new heat stress hotspots. Our hourly July analysis for France, shows that morning hours are the safest part of the day and that high heat stress can persist late into the afternoon hours. Mountain locations largely remain safe.

**Supplementary Information:**

The online version contains supplementary material available at 10.1038/s41598-025-30129-8.

## Introduction

Episodes of extreme heat have been increasing in frequency, magnitude and duration at many locations around the world, affecting human health and livelihoods^1–3^. Europe is no exception, with the 2022 summer season (June-July-August) being the hottest on record^4^, and the summers of 2019 and 2023 breaking multiple daily temperature records^5,6^. Extreme heat can affect human health in multiple negative ways, including heat exhaustion, heat related illnesses and death^7–10^.

While the extreme heat most severely affects vulnerable populations such as the elderly, infants, and people with pre-existing chronic conditions^8–10^, certain activities can be associated with a very high heat hazard increasing the risks of adverse heat effects in young and healthy adults too^11^. One such example is high endurance professional sporting events^12,13^. Firstly, exposure to heat is more dangerous in combination with increased physical activity, and particularly during high endurance activities^10,12–16^. Secondly, when challenged with the discomfort of overheating, the individual usually avoids the adverse effects through different behavioral responses such as seeking shade, reducing the intensity of the activity, or pausing to cool down. However, during sports competitions such individual protective behaviors are often ignored or may not always be implemented as needed^14^. In addition, exertional heatstroke, due to strenuous physical activity, can occur even without an exposure to high ambient temperatures^10^.

A number of major outdoor sporting events are held during the summer months in Europe with one of the largest being the Tour de France (TdF) - an annual multi-stage road bicycle race. First held in 1903, it is typically raced over the course of a 23 day period in July, predominantly in France but also in neighboring countries^17^. Recently, concerns have been raised regarding the impacts of climate change driven extreme heat episodes on major road cycling events, triggered by reports of riders suffering from heat related illnesses^18–20^. Racing in a Grand Tour such as the TdF places a significant physiological stress on its participants^21^. With an average of 21 stages over the three-week race, it has been argued that the TdF is the hardest endurance race in the world^22,23^. Thus, it is of little surprise that riders at high exertion levels will be more sensitive to changes in ambient conditions, and in particular heat^24,25^.

For many locations around the world, climate change has made the summer heat more uncomfortable and more dangerous for human health^26^. The mid-summer timing of the TdF race, and its long history of racing, provides a unique opportunity to study the changes in occurrence of extreme heat episodes experienced by the riders. We use the example of this major event to assess how the climatological heat stress has changed over the last 50 years and discuss the current heat safety protocols in road cycling and other high endurance sports.

Interestingly, the Giro d’Italia and Vuelta a España Grand Tours, despite taking place during the seasons that are climatologically less likely to encounter extreme heat (late spring and early autumn), have also experienced very hot days in recent years^20,27^. Heat safety and strategies for helping riders stay cool are rapidly gaining importance in Grand Tour cycling. From protecting the riders’ health to protecting their performance, the “art of staying cool” is expected to strongly influence the outcomes of future Grand Tour races.

## Methods

As a measure of heat stress, we use the Wet Bulb Globe Temperature (WBGT) - a physically-based heat index accounting for ambient air temperature, relative humidity, solar radiation and wind, which is also an international occupational health standard with clearly defined safety thresholds^28^. Some authors have suggested using the universal thermal climate index (UTCI) instead of WBGT in formulating sports safety guidelines^29,30^. However, their main criticisms appear to arise from the use of the simplified WBGT index that accounts for temperature and humidity only. Recent experiments testing the response of human participants to heat in laboratory conditions, have suggested that WBGT performs much better than the UTCI in predicting the human response to heat^31^. Moreover, the International Cycling Union (UCI)^37^ considers the WBGT in their current high temperature protocol, categorizing WBGT values below 15 °C as *very low-risk*, between 15 °C and 17.9 °C as *low-risk*, between 18 °C and 22.9 °C as *moderate low-risk*, between 23 °C and 27.9 °C as *moderate high-risk* and above 28 °C as *high-risk*.

**Fig. 1:**
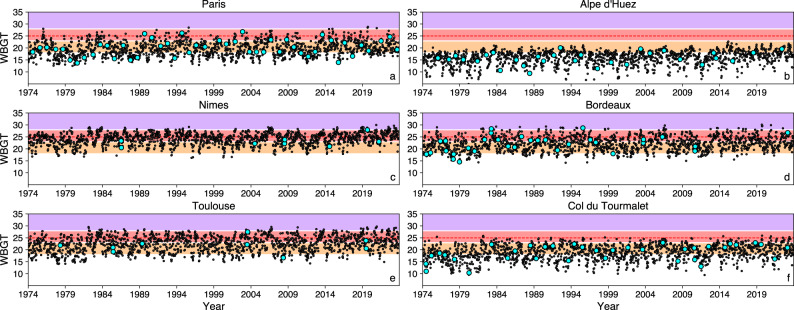
WBGT values at 1500 local time from 1974 to 2023 for every day in July (black dots) and on the dates of the Tour de France stage race at a given location (light blue dots). Shading indicates WBGT safety thresholds according to the UCI’s high temperature protocol^40^: 18 °C - 22.9 °C *moderate low-risk area* (orange); 23 °C - 27.9 °C *moderate high-risk area* (light red); > 28 °C *high-risk area* (purple). Official ISO WBGT safety threshold of 25 °C for very high exertion (metabolic rate above 520 W, acclimatized) is shown with dashed red line. Corresponding values at 1100, 1400, 1600, 1700 and 1800 h local time are shown in Figs. S1-S5. WBGT values for additional six TdF locations are shown in Fig. S6. WBGT values at 1500, 1600, 1700 and 1800 h on the actual race dates are also provided in Supplementary Tables S1- S6.

We calculate the WBGT using the thermofeel approach^32^, developed for use with gridded meteorological variables such as reanalysis products. It follows the original equation by Minard (1961),^33^ where WBGT is derived as a combination of dry bulb temperature (i.e., air temperature, Ta), wet bulb temperature, Tw, and globe thermometer temperature, Tg:

1 $$WBGT{\text{ }} = {\text{ }}0.7*Tw{\text{ }} + {\text{ }}0.2*Tg{\text{ }} + {\text{ }}0.1*Ta$$

In the thermofeel formulation, these terms are derived using 2-m air temperature, relative humidity, 10-m wind speed and mean radiant temperature (a measurement of incidence of radiation on a body). Specifically, wet bulb temperature is a function of air temperature and relative humidity, while globe thermometer temperature is a function of 2-m air temperature, 10-m wind speed and mean radiant temperature. This approach has been found to be accurate in comparison to classical WBGT calculations (Liljegren et al., 2008)^34^. Equations used in the thermofeel approach are described in detail in Brimicombe et al. (2023),^35^ more details are provided in the Supplementary Material.

We use hourly 2-m air temperature, dew point temperature and 10-m u and v wind components obtained from ERA5^36^, and mean radiant temperature from ERA5-HEAT data sets^37^. Both data sets are available on 0.25° x 0.25° (regular latitude x longitude) grids. Values at individual locations were obtained using the nearest neighbor remapping (CDO function “remapnn”)^38^. The relative humidity (RH), expressed in %, required for thermofeel calculation of WBGT, is calculated from the hourly values of 2-m air and dew point temperatures, Ta and Td, (expressed in degrees °C) using the revised Magnus equation. ^39^ (see Supplementary Material for details).

The meteorological conditions faced by riders during a given stage of a race are clearly not constant. However, in the absence of data describing the exact conditions experienced by an individual rider during the race, we consider the meteorological conditions at given geographical locations, selecting several places that are more commonly visited during the various stages of the Tour de France. We first show the calculated WBGT values at six locations, the cities of Paris, Nîmes, Bordeaux and Toulouse and the mountain locations of Alpe d’ Huez and Col du Tourmalet. These locations were chosen because they have a history of race visits during the period considered and provide a mix of climate types (e.g., continental, mountainous, humid, dry). In the Supplementary Material, we complement the analysis by adding heat stress values for an additional six locations (Marseille, Nice, Montpellier, Grenoble, Lyon and Pau). For the selected locations, we calculate the hourly WBGT values for each day in July from 1974 to 2023 and highlight the dates when the race visited them. While a given location may exhibit a positive trend in heat stress over the considered 50-year period, it is still possible for the race dates to fall on cooler days and to not be apparently affected by these changes. For this reason, we chose to show both the values for the race dates and also for each day in July and compare them. It should be noted that the time of day any of these locations are actually visited by the peloton on different race dates may vary. We emphasize the 1500 h local time WBGT values as one of the climatologically highest heat stress hours during which the race takes place. In the Supplementary Material, we additionally show the heat stress values for 1100, 1400, 1600, 1700 and 1800 h local time. As stated above, in the absence of available public data about the exact meteorological conditions ‘following the riders’ during the course of the race, we instead focus on understanding the temporal changes in heat stress over the fixed points during the last 50 years and discuss any changes in riding conditions. While this approach allows us to understand trends and changes in the occurrence of extreme heat episodes in a particular location, it is important to note that the dynamic rider exposure is more complex than this and will be constantly impacted by different microclimatic effects along the route (e.g., being in a shaded or sun-exposed area, protected from or exposed to the wind, passing by cooler tree-covered areas or heat trapping urban areas).

**Fig. 2:**
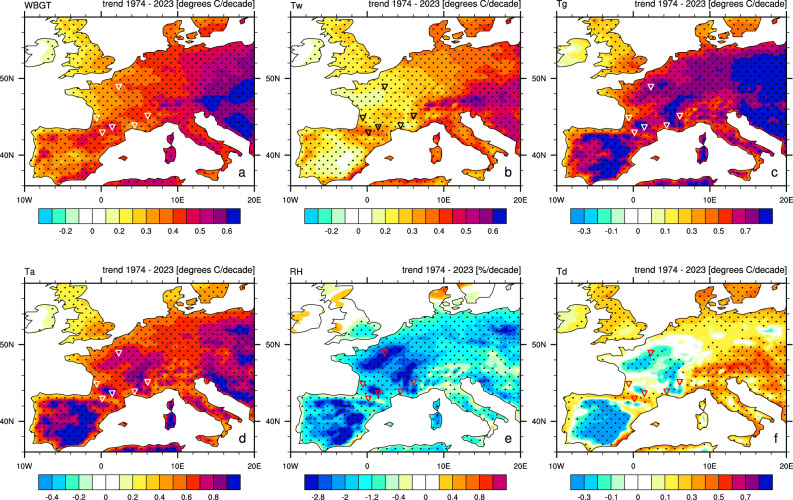
Trends in 1500 h local time July WBGT (**a**), wet bulb temperature Tw (**b**), globe temperature Tg (**c**), air temperature T_a_ (**d**), relative humidity RH (**e**) and dew point temperature Td (**f**) over a period from 1974 to 2023. Dotted regions indicate trends that are significant at 95% confidence level. _a_. WBGT, Ta and RH July trends at 1100, 1300 and 1700 h local time are shown in Fig. S7.

**Fig. 3:**
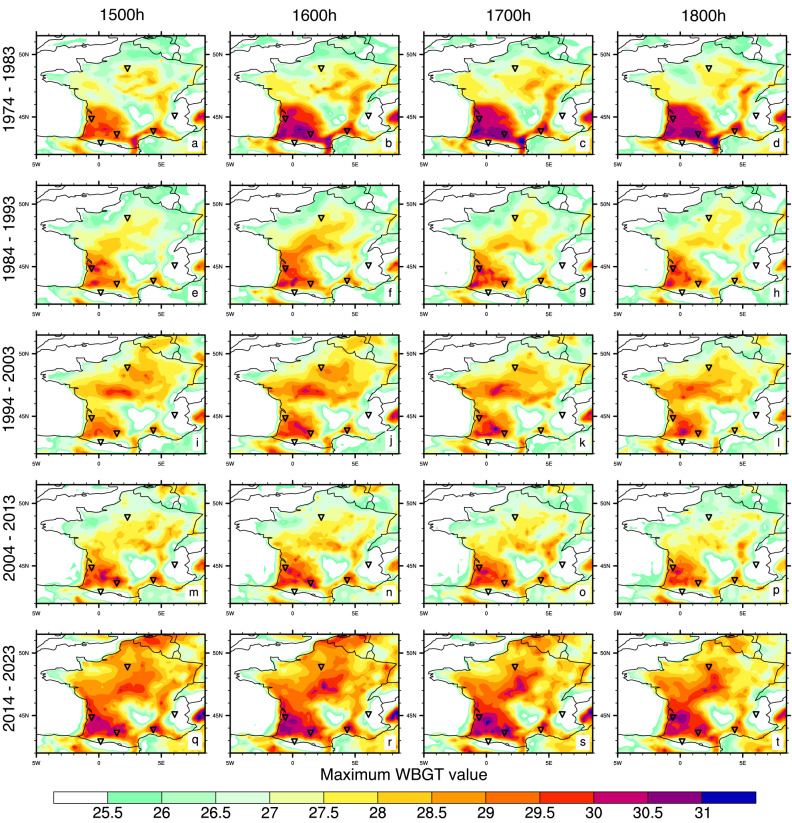
Maximum WBGT values [°C] at 1500, 1600, 1700 and 1800 h local time, for each decade since 1974: (**a**-**d**) 1974–1983; (**e**-**h**) 1984–1993; (**i**-**l**) 1994–2003; (**m**-**p**) 2004–2013 and (**q**-**t**) 2014–2023. Corresponding values for 1100, 1200, 1300 and 1400 h local time are shown in Fig. S8.

**Fig. 4:**
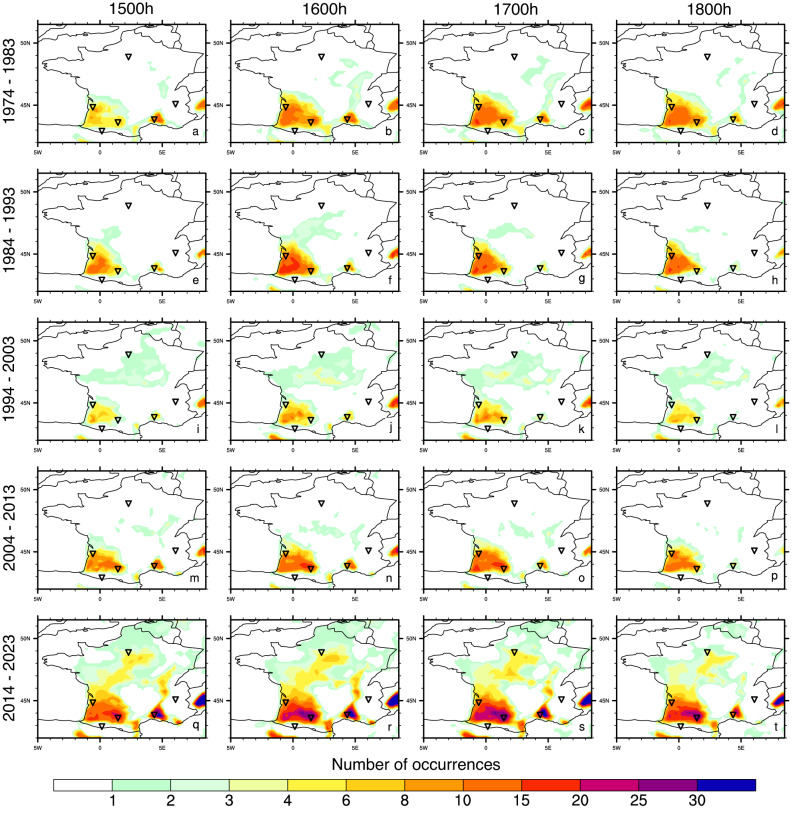
Number of occurrences of crossing the UCI’s high-risk WBGT threshold of 28 °C at 1500, 1600, 1700 and 1800 h local time, for each decade since 1974: (**a**-**d**) 1974–1983; (**e**-**h**) 1984–1993; (**i**-**l**) 1994–2003; (**m**-**p**) 2004–2013 and (**q**-**t**) 2014–2023. Corresponding values for 1100, 1200, 1300 and 1400 h local time are shown in Fig. S9.

We then turn to mapping the heat stress changes across all of Metropolitan France (Figs. 2, 3 and 4 and Figs. S7-S9). In Fig. 2 (and Fig. S7), we show 50-year trends in WBGT and its cofounding variables; in Fig. 3 (and Fig. S8) we show the maximum WBGT values for each decade at different times of day; and in Fig. 4 (and Fig. S9) we show the number of occurrences of crossing the high-risk WBGT threshold of 28 °C, for each decade at different times of day. While the consideration of future climate projections is outside the scope of the current study, the climate data presented in Figs. 3 and 4 and Figs. S8 and S9, provides a baseline for future events planning, both in respect to historically high-risk areas and in regards to the safest times of the day.

## Results

### Heat stress from 1974 to 2023

The black dots in Fig. 1 (and in Figs. S1-S5) show the WBGT values at 1500 h (and 1100, 1400, 1600, 1700, and 1800 h, respectively) local time for each day in July for Paris, Nîmes, Bordeaux, Toulouse, Alpe d’Huez and the Col du Tourmalet. For five out of the six locations, the highest WBGT occurrences at 1500 h since 1974 have all been recorded post 2018: Bordeaux (30.1 °C WBGT in 2019), Nîmes (30.0 °C WBGT in 2020), Toulouse (29.7 °C WBGT in 2020), Paris (28.8 °C WBGT in 2019) and the Col du Tourmalet (25.9 °C WBGT in 2019); see Fig. 1. The highest value for Alpe d’Huez was 22.7 °C WBGT, occurring in 2015. Excluding the two mountainous locations, each of the above mentioned WBGT records would fall well in the *high*-*risk* category according to the UCI’s extreme heat protocol (WBGT > 28 °C).

Fortunately for the Tour de France riders, organizers and spectators, the actual dates of the TdF race have so far avoided the worst of the heat. For Paris, the highest WBGT value on the day of a TdF stage was 26.8 °C, in 2002 (recorded both at 1500 h and 1600 h local time; Fig. 1 and Fig. S3, Table S1). Since 1974, the WBGT high-risk threshold of 28 °C was crossed on 5 occasions in Paris in July at 1500 h (and also at 1600 h), but this has so far not coincided with TdF dates (Fig. 1). For the other five locations, the highest WBGT occurrences on race dates were: 20.7 °C for Alpe d’Huez in 1992 at 1700 h; 27.9 °C for Nîmes in 2019 at 1400 h and 1500 h; 29.2 °C in Bordeaux in 1995 at 1600 h; 27.9 °C for Toulouse in 2003 at 1800 h; and 24.2 °C for Col du Tourmalet in 1983 at 1700 h (Fig. 1 and Figs. S3-S5, Tables S1-S6). Comparison of the timeseries of WBGT values at 1100, 1400, 1600, 1700 and 1800 h local time (Figs. S1-S5), suggest that the heat stress is lowest at 1100 h local time, and depending on the location, can remain high even at 1800 h local time. Consideration of additional locations (Figure S6) indicates Pau and Lyon as locations where crossing the *high-risk* limit of 28 °C WBGT is not uncommon. The 1995 Tour de France stage through Pau (Fig. S6) occurred on a day when the 28 °C WBGT *high-risk* threshold was exceeded at 1600 h local time.

### Trends in heat stress and its components from 1974 to 2023

The WBGT trends over the entire Metropolitan France are positive, demonstrating an increase in afternoon heat stress values in July over the 50-year period (Fig. 2 a and Fig. S7). The trends in WBGT are lowest in the very northwest of the country (0.1 °C WBGT/decade) and increase moving to the east or south. The largest trends, exceeding 0.5 °C WBGT/decade are seen in the southern (Occitanie) and eastern parts of France (Auvergne-Rhône-Alpes and Bourgogne-Franche-Comté). Although not commonly visited by the TdF, notable positive trends are seen throughout the eastern coast of Spain, Italy, Switzerland and the Adriatic coast of Croatia. Typically, these exceed 0.4 °C WBGT/decade and in some locations are even larger than 0.6 °C WBGT/decade.

Differences in the geographical distribution and magnitudes of WBGT trends can be understood by considering the trends in its main components: Tw, Tg and Ta (Fig. 2a–d). For the period considered, positive WBGT trends (Fig. 2a) are present over all of Europe, although they are lower over western areas compared to eastern and central Europe. The wet bulb temperature trends show a similar pattern with larger increases over eastern Europe (Fig. 2b). In contrast, globe thermometer temperature and air temperature show the highest increase over central Spain and France, Sicily and eastern Europe. (Fig. 2c and d). It is not a surprise that WBGT trends are dominated by the trends in Tw. From Eq. 1, it is clear that between its three components (Ta, Tw and Tg), changes in Tw will have the largest impact on WBGT due to the size of the multiplying coefficient.

In the thermofeel formulation used^33^, the wet bulb temperature is derived from air temperature and relative humidity. The relative humidity displays significant negative trends almost everywhere, peaking over the central parts of France and Spain (Fig. 2e). This explains the lower trends in Tw, and consequently WBGT, over these areas. It should be noted that the negative correlation between sub-daily air temperature and relative humidity is a very common occurrence. This is because at higher temperatures the air can hold more water and thus in the absence of additional sources of atmospheric moisture, the actual water content of air (the specific humidity) can remain the same while the relative humidity will decrease.

From a heat stress (i.e., WBGT) perspective, over the central parts of France and Spain, the increase in temperature (positive trend > 0.6 °C per decade) is more strongly offset by the decrease in relative humidity (negative trend > 2% per decade), leading to an overall weaker increase in WBGT compared to the central and eastern Europe where the humidity decrease is much weaker. Relative humidity is closely related to dew point temperature which is perhaps a more intuitive measure of how uncomfortable ambient humidity feels: higher dew point temperature equates to more moisture in the air and thus more discomfort. In panel 2f we see that the dew point temperature displays positive trends everywhere except over the central parts of France and Spain. The fact that the temperature increase is not accompanied by a dew point temperature increase over these areas is helpful for dampening the resulting heat stress changes.

### Geographical distribution of maximum heat stress values and the frequency of threshold crossings

In Figs. 3 and 4, we show the maximum WBGT values and number of occurrences of crossing the UCI’s *high-risk* WBGT threshold of 28 °C for each decade since 1974. While Figs. 3 and 4 focus on the afternoon hours (1500, 1600, 1700 and 1800 h local time), in Figs. S8 and S9 we show the same for the close to midday values (1100, 1200, 1300 and 1400 h local time). The maximum WBGT values peak in southwest of France with values above 30 and 31 °C WBGT recorded at several locations (Fig. 3). However, in the most recent decade, 31 °C WBGT has also been reached in central France, a region with the potential to become a new hotspot for extreme heat stress. Comparison of Fig. 3 and Fig. S8, shows that the maximum recorded heat stress values in July increase throughout the day, peaking between 1500 and 1800 h local time. The number of occurrences of crossing the high-risk thresholds have increased in more recent decades, both in known hotspots in southwest France and the Rhône delta^26^, but also over new ones in the Rhône valley and central France (Fig. 4). In the most recent decade, the majority of France has encountered at least one occurrence of crossing this threshold. The high-risk thresholds are more often crossed in the late (1500–1800 h) compared to the early (1200–1400 h) afternoon while the late morning hours (1100 h) show almost no occurrences (Fig. 4 and Fig. S9).

## Discussion

### Protocols for extreme heat

In 2023, the International Cycling Union (UCI) adopted a new High Temperature Protocol^40^ for riders’ safety that accounts for the impacts of several meteorological factors through the use of WBGT. Many other major governing bodies in sports, including the International Tennis Federation (ITF), the Fédération Internationale de Football Association (FIFA), the Fédération Internationale de Volleyball (FIVB), World Triathlon, World Athletics, and World Rowing all currently require the use of WBGT to assess heat safety^16,41–44^. After a history of using air temperature alone to define when high heat may threaten athletes’ safety, the inclusion of heat stress indicators such as WBGT by different sports authorities is a welcome step forward for most sports (with the exception of disciplines where heat stress is less of an issue, e.g., hockey or winter sports). However, issues with the lack of meteorological measurements needed for accurate WBGT calculation have been reported, resulting in sub-optimal representation of climatological heat stress through the use of simplified WBGT versions^29,30^. It is also important to note that different international federations propose different WBGT thresholds in their safety protocols. The UCI currently employs a WBGT *high-risk* threshold of 28 °C, while the ITF uses two higher thresholds of 30.1 °C and 32.2°C^16^. For WBGT above 28 °C, the UCI recommends actions such as moving start zones to shaded areas, moving start times or postponing sections of races^16,40^. At a WBGT of 30.1 °C, the ITF invokes the ‘modification of play’, allowing for one extra 10-minute break between the second and third sets of singles matches. A WBGT value of 32.2 °C represents ITF’s guideline value for the suspension of play, although other factors such as the weather during the previous two days are also considered^16^. FIFA and World Rowing use a WBGT threshold of 32 °C to introduce mandatory cooling breaks or invoke cancellations^16^. The WBGT threshold of 32 °C used by several governing sports authorities is surprisingly close to the International Organization for Standardization (ISO) WBGT resting rate limit of 33 °C^28^, and could potentially be too high for sports activities that feature long periods of exertion and high metabolic rates.

To the authors’ knowledge, there is currently not enough *publicly* available, individual sport specific data describing the actual human response to heat that would allow derivation of tailored thresholds and heat stress indicators for a given sport. Comprehensive thresholds based on the actual human responses to heat were derived in the late 1950 s, with the goal of protecting the US Army and Marine Corps from heat related illness during training exercises^45^. Based on these, the International Organization for Standardization (ISO) defined the acclimatized WBGT safety thresholds of 25 °C for high exertion (metabolic rate above 520 W), 28 °C for moderate metabolic rate (> 300 W) and 33 °C for resting metabolic rate (< 115 W)^28^. Clearly, the conditions experienced by athletes and soldiers can differ in many ways, including factors such as the type of clothing, metabolic rates, duration of activity, access to hydration or previous acclimatization. The most adequate sports-based thresholds will depend on careful evaluation of the activity and any limitations on physical cooling processes such as sweating. Thus, although the UCI classification of a WBGT value of 28°C as *high risk* is higher compared to the ISO value of 25 °C, this may be appropriate when considering factors such as thin sports clothing, periods of reduced exertion on downhill sections, or additional cooling resulting from high racing speeds. It is also useful to note that very high core body temperatures have been recorded in elite cyclists during time trials and road racing, with one study showing that 85% of the athletes monitored reached a core temperature of at least 39 °C and 25% exceeded 40°C^25^. This suggests that professional athletes are likely used to withstanding higher levels of heat stress compared to the rest of the population and this should be considered in safety threshold definitions.

### Extreme heat mitigation strategies

Current protocols used by major sport bodies such as the International Olympic Committee (IOC) are based on a Delphi expert process^46^. Such an expert review process is necessary because there is no definite data-based answer as to which exact critical environmental limits affect the athletes physiologically. Previous studies have however provided some information on critical environmental ranges where additional vigilance should be exercised. Using the data from a self-paced cycling time trial, Faulkner et al. (2025)^47^ found performance impairment for temperatures above 20 °C. They suggest that the individual thresholds most likely lie between 20 and 30 °C and that above these temperatures, pre‐ and per‐cooling strategies should be implemented in order to mitigate negative impacts on endurance performance. Periard et al. (2023)^48^ studied the relationship between the thermal and cardiovascular strain of professional cyclists during a UCI World Tour multistage cycling race (the 2019 Tour Down Under). The variables found to be the most strongly correlated with mean power output across the different stages were WBGT, globe temperature (Tg), UTCI and relative humidity. The highest core body temperature values were measured on temperate (~ 23 °C), warm (~ 28 °C), and hot (~ 36 °C) days, confirming that core temperature during multistage racing is associated with multiple environmental factors other than the ambient temperature. Junge et al. (2016)^49^ suggested that for outdoor cycling, acclimatized subjects may maintain average time-trial speed and performance under conditions as high as 28 °C WBGT. This is in line with the current UCI extreme temperature protocol. When considering moderate cycling intensity in laboratory conditions, Bogaard et al. (2025)^50^ found that mean critical wet-bulb globe temperature, after heat acclimatization, can exceed 30 °C WBGT in some cases. They suggested that heat acclimation can increase critical WBGT limits by 2.3 ± 2.7 °C in warm-humid conditions, with more than 5 days of heat acclimation necessary.

For cycling specifically, an additional factor to consider is that by moving through the air at velocities that can often go above 50 km/h^22^, riders are exposed to an ‘apparent’ wind^51,52^. The expectation is that for ambient temperatures that are lower than human body temperature, this would have a beneficial cooling effect on an otherwise warm day, both in terms of convective and evaporative cooling. However, at ambient temperatures approaching human body temperature or higher, the exchange of heat with the surrounding environment may be affected^53,54^. For example, at air temperatures higher than skin temperature, the process of exchange of heat with the surrounding air by convection will no longer result in cooling. Depending on other meteorological and physiological factors (e.g., humidity, sweating rates) an increase in evaporative cooling may not be able to counteract this. The interaction between the apparent wind and perceived heat stress is not accounted for by the standard heat stress indicators because those are based on the actual, observed wind and not the one experienced by a person moving at high speed. The impacts of apparent wind on dissipation of heat and sweating rates, especially at air temperatures above normal human body temperatures, require deeper understanding in order to be implemented into extreme weather protocols.

Current mitigation strategies for road cycling are diverse and include: warm-up in the shade with fans (for WBGT in the range 15–17.9 °C); warm-up with ice vests, use of wet towels, application of individualized hydration plans, distribution of ‘ice-socks’ and supply of ice during the race (for WBGT in the range 18 °C – 22.9°C); adaptation of the start area to keep riders in the shade before the start, protection of non-athlete staff from the sun, increase in the number of motorbikes providing riders with drinks and ice packs, adaptation of the rules limiting hydration and cooling in competition (for WBGT in the range 23 °C – 27.9°C) and finally, modification of start and finish times, or route or cancellation when the environmental conditions exceed 28 °C WBGT^16,40^.

Developing the optimal cooling strategies requires constant monitoring and re-evaluation^55^. For example, Lynch et al. (2017)^56^ found that the currently recommended fan use intervention at the Australian Tennis Open can be improved if applied in combination with skin wetting. This combined intervention is reported to reduce the mean skin temperature by ∼1.0–1.5 °C while mitigating rises in heart rate. Brown et al. (2024a and 2024b)^57,58^ evaluated the effectiveness of FIFA cooling break policies at 32 °C WBGT in laboratory conditions. They found that the current policies involving 3-minute cooling breaks with chilled fluid ingestion and ice towels, attenuated thermal, cardiovascular and perceptual strain in male participants^57^ but not in female participants^58^. The addition of 5 min to the half-time break combined with the 3-minute cooling break (an approach not currently applied) was shown to effectively mitigate cardiovascular and heat strain in females^58^.

### Towards tailored heat stress indicators

One major limitation in assessing the impact of heat stress on different sports is the lack of physiological and sometimes even meteorological data experienced by the athletes. To help the development of tailored heat stress indices for improved safety protocols, we appeal to both teams and organizers of different sport events to share with the scientific community anonymized data on participants’ physiological response to heat and any available local meteorological measurements^59,60^. Different sports should not be expected employ the same heat stress thresholds^61^, but more publicly available data is needed to understand the interplay between exertion, duration and other compounding factors affecting athletes’ heat safety. During the 2020 Tokyo Summer Olympics, the incidence rate for heat related illnesses reached a remarkable 14% for sport disciplines like the marathon and race-walking^62^. The 2024 Paris Summer Olympics had the athletes, organizers and spectators experiencing extreme heat yet again^63^, reminding us of the urgency of this issue. Further research is needed to move towards tailored heat stress indicators for individual disciplines. One promising way is by adopting a human heat balance approaches that would take into account environmental and physiological aspects as well as information about exertion to derive the critical environmental limits at which a safe core temperature can no longer be maintained.

## Conclusions

In the field of sports medicine, several recent works have provided comprehensive recommendations for sports authorities concerning cooling techniques, changes to regulatory limitations for hydration and cooling, adaptation of distances and durations, adaptation of surfaces to increase the albedo and built material for increase in shaded area, environmental monitoring of historical data before making decisions on future events, etc^13,15,16,21,46,61,65^. We reiterate all of these and specifically, for future Tour de France editions, recommend continued development and re-evaluation of hot weather emergency protocols; mobile measurements of meteorological conditions experienced while riding at race speeds accompanied by stationary measurements along the race path; measurements of riders’ core body temperatures and other physiological parameters such as sweating rates^66,67^; and finally, education of riders and their supporting teams, as well as spectators, about heat safety and the signs of heat-related illness. It is interesting that the Tour de France race dates have thus far managed to avoid the worst of the July heat stress. However, given that the route and the race dates have to be planned months in advance, while reliable weather forecasts are available maximum 14 days beforehand^68^, this outcome is apparently by chance. Accordingly, it is critical that both organizers and participants (and to a lesser extent, the spectators) remain vigilant and prepared. In the absence of detailed daily weather forecasts several months before the event, awareness of the locations with a history of dangerous heat stress occurrences as well as emerging ones, is of key importance.

We summarize our conclusions as follows: (i) July WBGT values for France show positive trends over the last five decades (Fig. 2a), both the magnitude and number of occurrences of high heat stress events have increased over recent decades (Figs. 3 and 4); (ii) the areas around Toulouse, Pau and Bordeaux in France’s southwest, and also around Perpignan and Nîmes in the southeast have a history of high heat stress episodes, extra caution should be exercised when planning the events in these regions; (iii) new heat stress hotspots are emerging, with locations like Paris and areas across central France starting to cross the UCI’s *high-risk* WBGT threshold of 28 °C more commonly (Figs. 1 and 4, Figs. S6 and S8); (iv) from a climatological viewpoint, in July in France, morning hours are the safest part of the day (Figs. S8 and S9), while high heat stress can persist during most of the afternoon (Figs. 3 and 4), planning the race for the morning hours and avoiding the afternoons could substantially increase rider and spectator safety; (v) mountain locations largely remain within low-risk and moderate low-risk WBGT values throughout the day (for now).

## Supplementary Information

Below is the link to the electronic supplementary material.

Supplementary Material 1

## Data Availability

Thermofeel code for calculating WBGT is available at: [https://doi.org/10.21957/mp6v-fd16](https:/doi.org/10.21957/mp6v-fd16). The climate variables used for WBGT calculations are freely available from Copernicus Climate Data Store at: [https://cds.climate.copernicus.eu/datasets/reanalysis-era5-single-levels-timeseries? tab=overview](https:/cds.climate.copernicus.eu/datasets/reanalysis-era5-single-levels-timeseries? tab=overview) and https://cds.climate.copernicus.eu/datasets/derived-utci-historical? tab=overview.
