# Supplementary material for: The future of European outdoor summer sports through the lens of 50 years of the Tour de France
Source: Sci Rep. 2026 Feb 24;16:2644. doi: 10.1038/s41598-025-30129-8 (PMC12932698; doi:10.1038/s41598-025-30129-8)
Supplement: Supplementary file 1 — Supplementary Material 1 [file 41598_2025_30129_MOESM1_ESM.pdf]

Supplementary Material for “The future of European outdoor summer sports through the lens of 50 years of the Tour de France“ by I. Cvijanovic et al.

Contains: Supplementary Methods, Supplementary Figures S1 - S9, Supplementary Tables S1 - S6.

Supplementary Methods

Thermofeel code

Equations used in the thermofeel approach are described in detail in Brimicombe et al. (2023)[1], the code is openly available at: https://github.com/ecmwf/thermofeel/blob/master/thermofeel/thermofeel.py#L601).

Relative humidity calculation

The relative humidity (RH), expressed in %, required for thermofeel calculation of WBGT, is calculated from the hourly values of 2-m air and dew point temperatures, Ta and Td, (expressed in degrees ° C) using the revised Magnus coefficients (following Lawrence et al. 2005[2]):

RH = 100*exp(17.625 * (Td/ (243.04 + Td) - Ta/ (243.04 + Ta)))   (1)

References:

(1) Brimicombe C, et al. Wet Bulb Globe Temperature: Indicating Extreme Heat Risk on a Global Grid. Geohealth 2023; 7. DOI: 10.1029/2022GH000701.

(2) Lawrence MG. The Relationship between Relative Humidity and the Dewpoint Temperature in Moist Air: A Simple Conversion and Applications. *Bull Amer Meteor Soc* 2005; **86**: 225–34.

Supplementary Figures

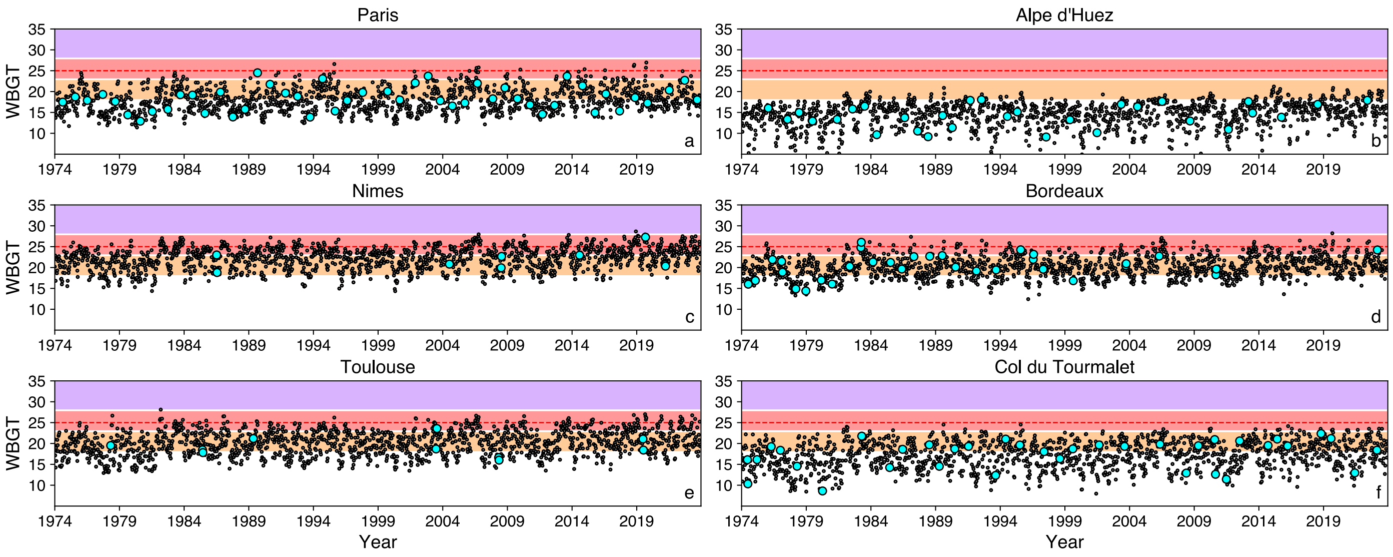

Fig. S1: As in Fig. 1 but for 1100 h local time.

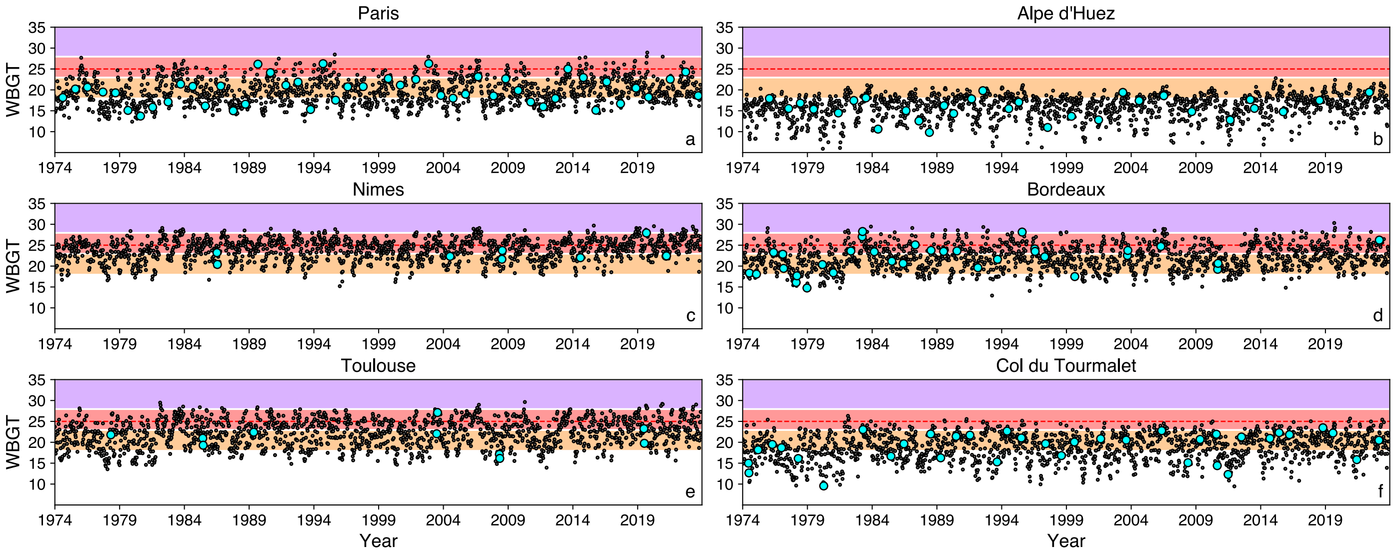

Fig. S2: As in Fig. S1 but for 1400 h local time.

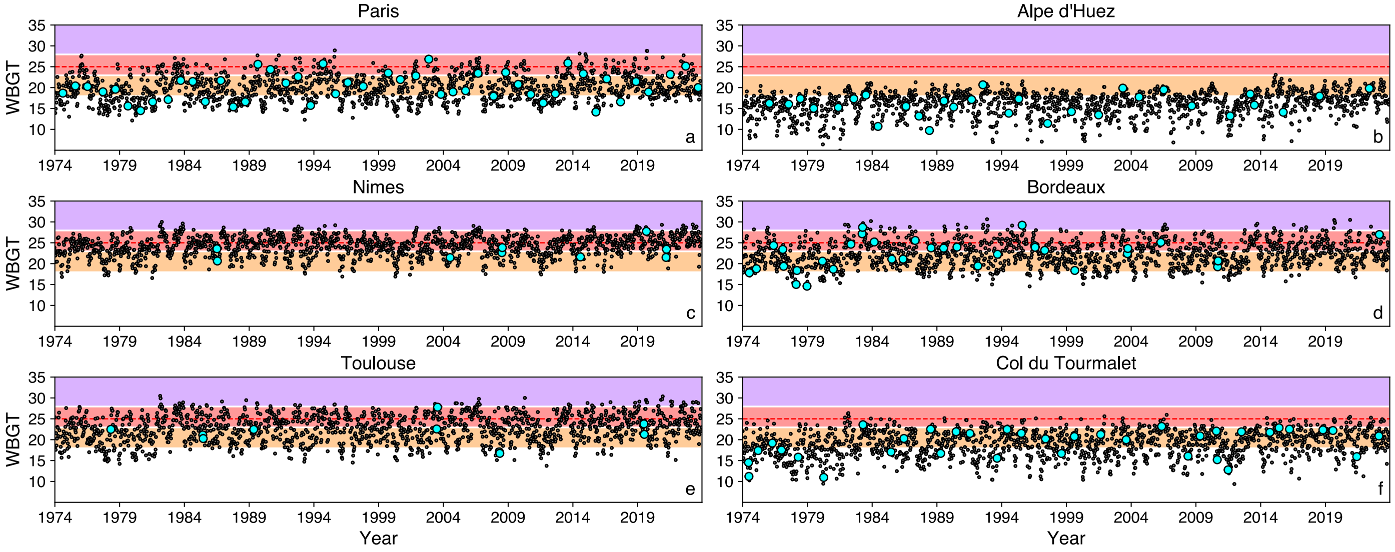

Fig. S3: As in Fig. S1 but for 1600 h local time. WBGT values at 1600 h on the dates of the Tour de France stage race are also displayed in Supplementary Tables S1-S6.

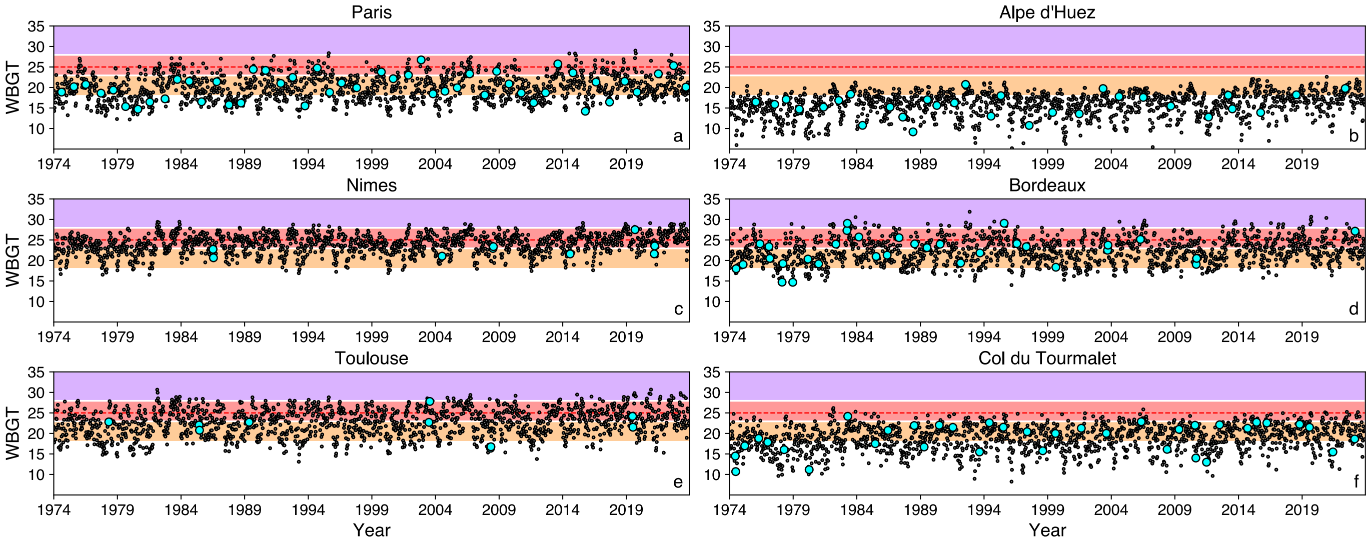

Fig. S4: As in Fig. S1 but for 1700 h local time. WBGT values at 1700 h on the dates of the Tour de France stage race are also displayed in Supplementary Tables S1-S6.

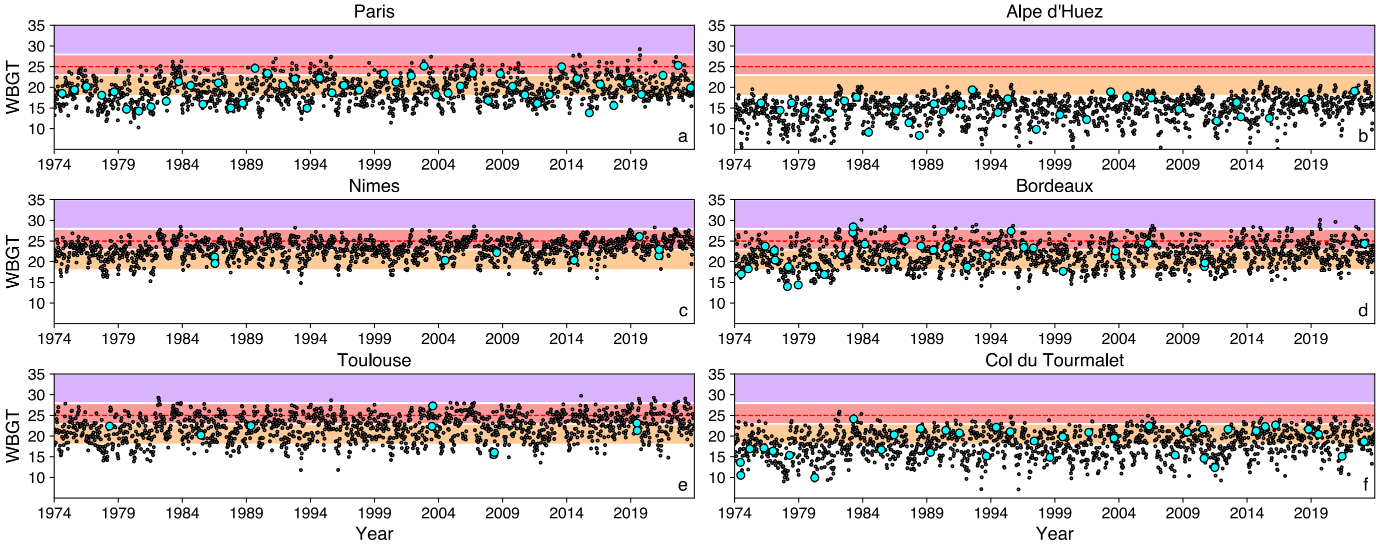

Fig. S5: As in Fig. S1 but for 1800 h local time. WBGT values at 1800 h on the dates of the Tour de France stage race are also displayed in Supplementary Tables S1-S6.

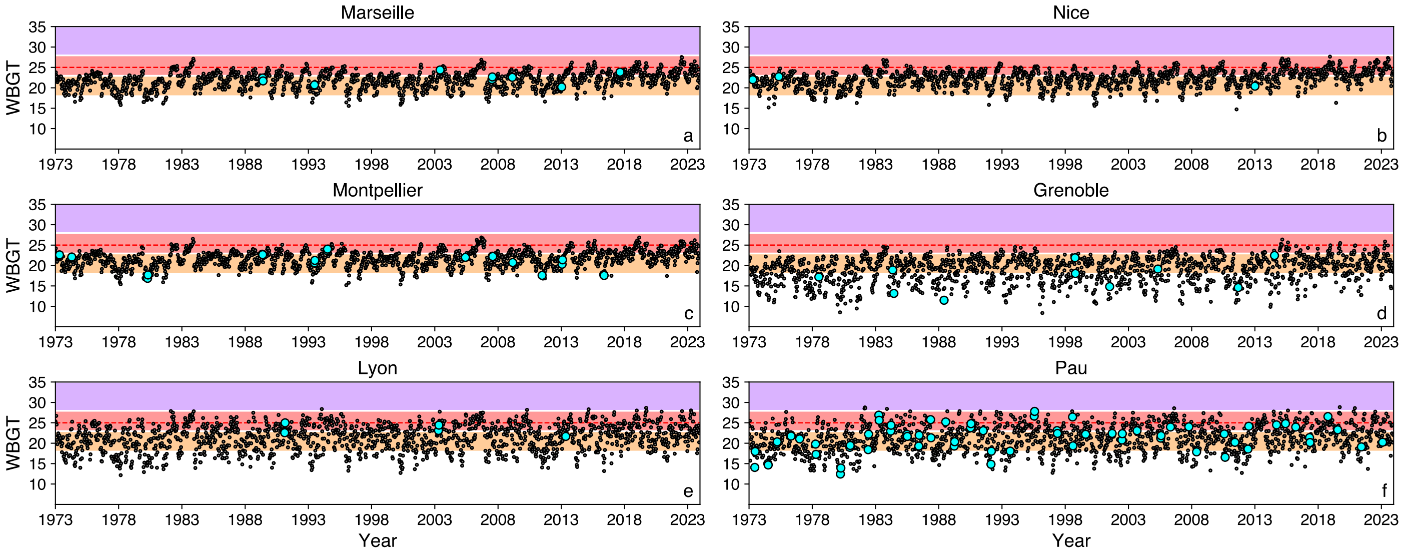

Fig. S6: WBGT values at 1500 h local time from 1973 to 2023 for every day in July but for Marseille (a), Nice (b), Montpellier (c), Grenoble (d), Lyon (e) and Pau (f). Shading indicates WBGT safety thresholds according to the UCI's high temperature protocol: 18°C - 22.9°C *moderate low-risk area* (orange); 23°C - 27.9°C *moderate high-risk area* (light red); > 28°C *high-risk area* (purple). Official ISO WBGT safety threshold of 25°C for high exertion (metabolic rate above 520 W, acclimatized) is shown with dashed red line.

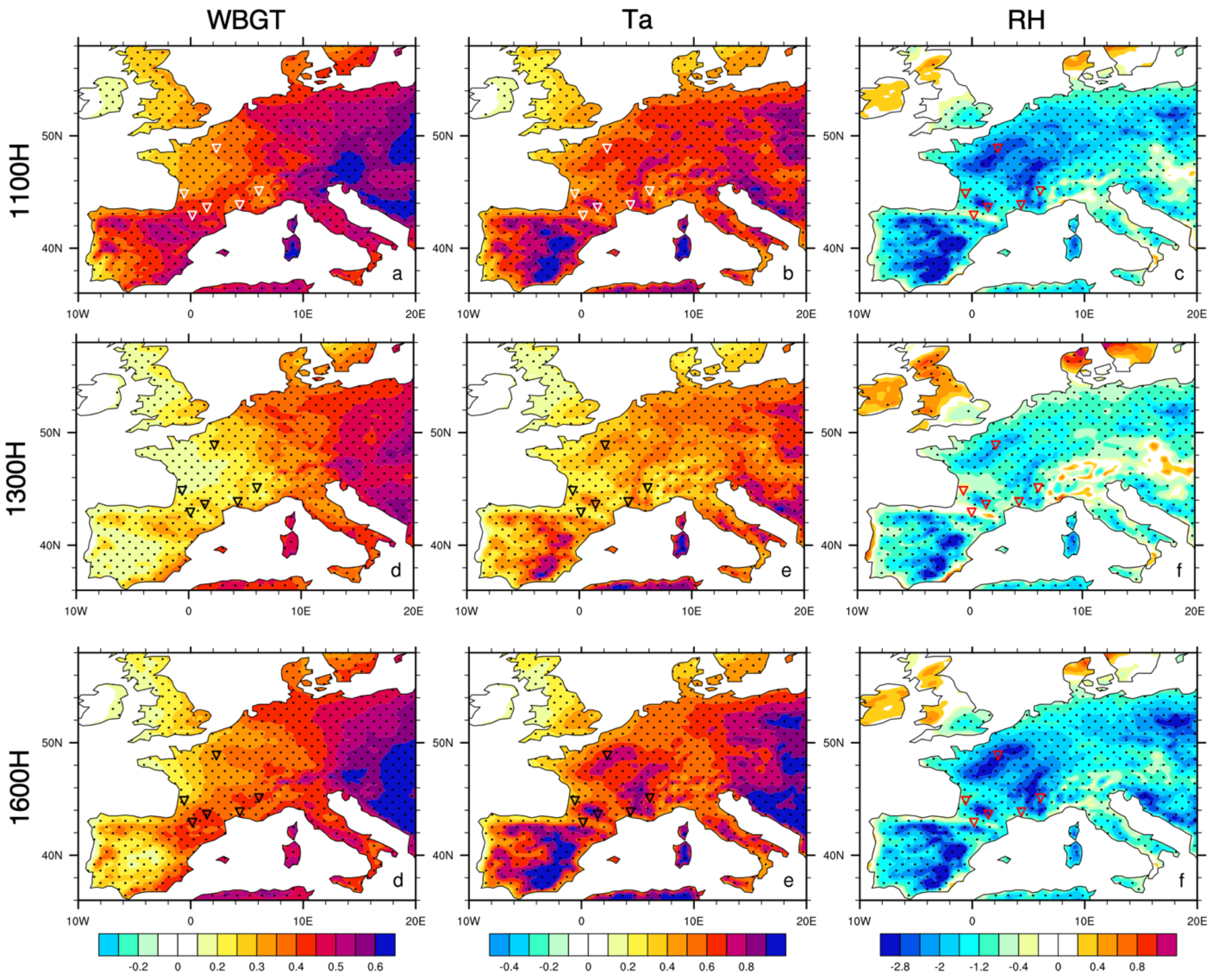

Fig. S7: Trends in July WBGT [°C / decade] (a-d), air temperature T_a [°C / decade] (e-h) and relative humidity RH [% / decade] (i-l) at 1100, 1300 and 1600 h local time over a period from 1974 to 2023. Dotted regions indicate trends that are significant at 95% confidence level.

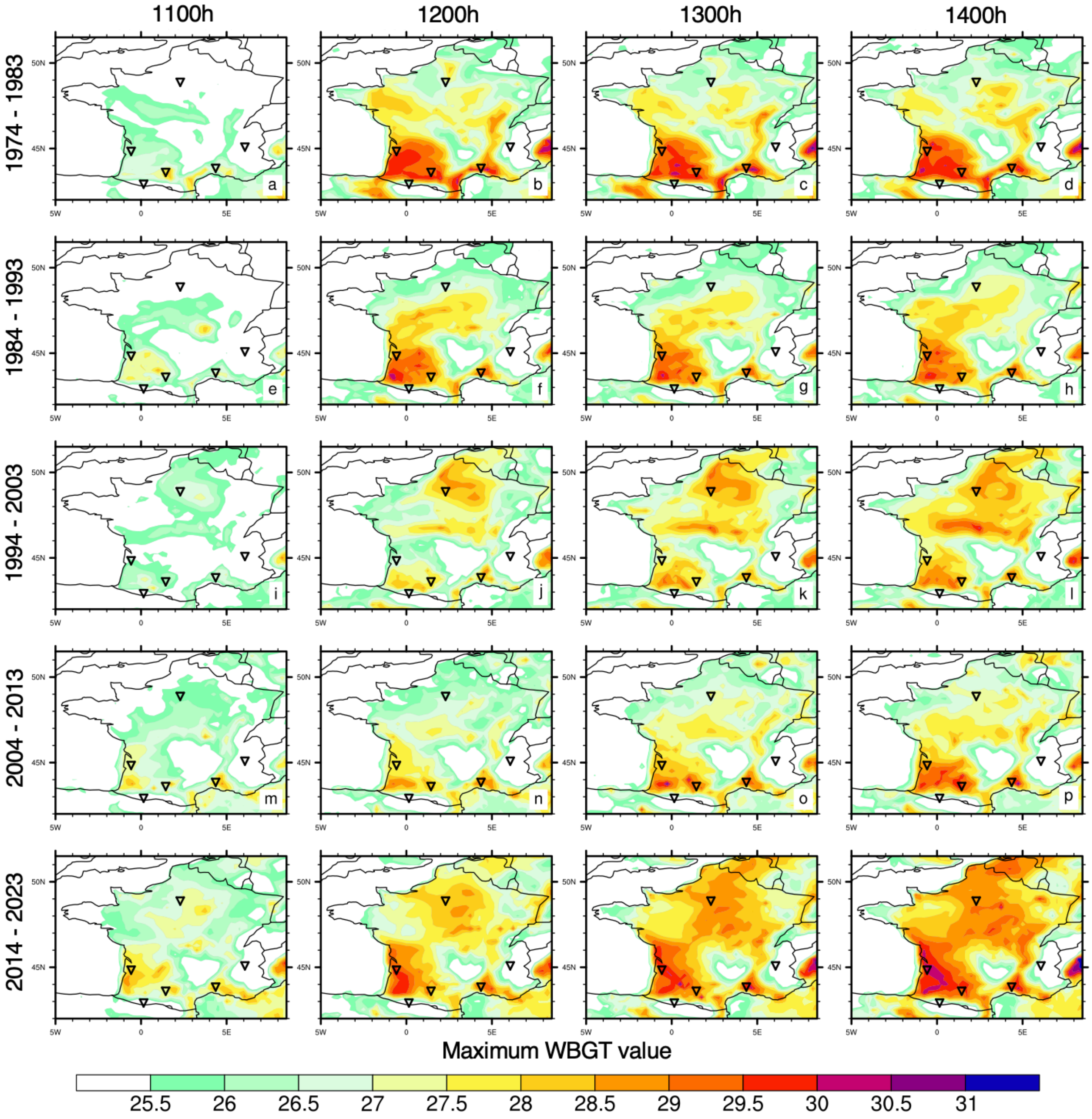

Fig. S8: Maximum WBGT value [°C] at 1100, 1200, 1300 and 1400 h local time, for each decade since 1974: a-d) 1974-1983; e-h) 1984-1993; i-l) 1994-2003; m-p) 2004-2013 and q-t) 2014-2023.

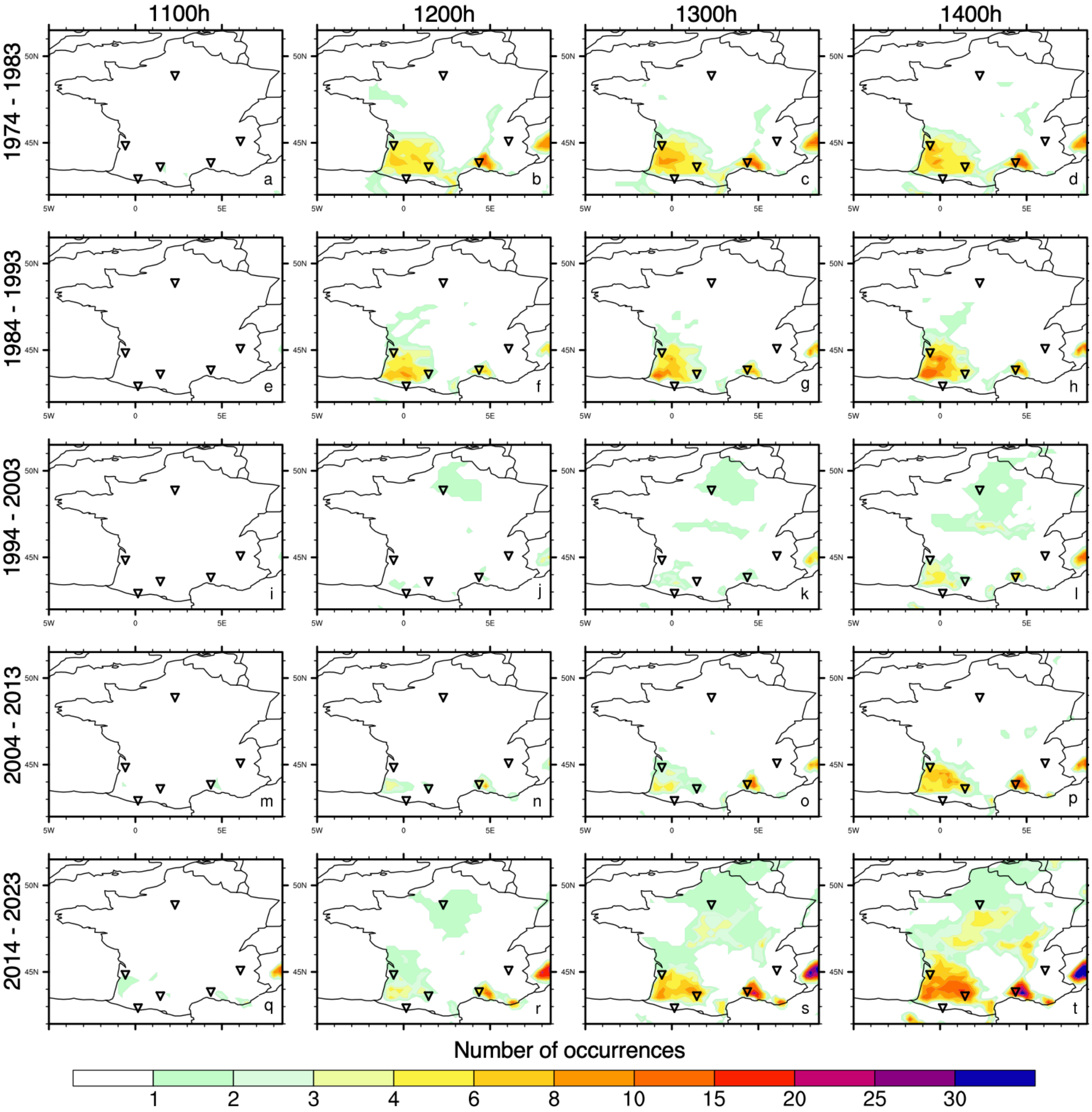

Fig. S9: Number of occurrences of crossing the UCI's high risk WBGT threshold of 28 °C at 1100, 1200, 1300 and 1400 h local time, for each decade since 1974: a-d) 1974-1983; e-h) 1984-1993; i-l) 1994-2003; m-p) 2004-2013 and q-t) 2014-2023.

Supplementary Tables

Supplementary Table S1: WBGT values [°C] at 1500, 1600, 1700 and 1800 h local time on the dates of the Tour de France stage race through Paris

Date	WBGT 1500h	WBGT 1600h	WBGT 1700h	WBGT 1800h
**1974-07-21**	18.31	18.68	18.88	18.91
**1975-07-20**	20.11	20.41	20.19	19.86
**1976-07-18**	20.23	20.26	20.74	20.57
**1977-07-24**	19.45	19.00	18.62	18.64
**1978-07-23**	19.49	19.61	19.38	19.24
**1979-07-22**	14.94	15.60	15.36	15.23
**1980-07-21**	13.71	14.48	14.71	14.40
**1981-07-19**	15.96	16.59	16.45	16.21
**1982-07-25**	17.20	17.13	17.23	17.36
**1983-07-24**	21.41	21.71	21.97	21.84
**1984-07-22**	20.95	21.46	21.51	21.31
**1985-07-21**	15.51	16.64	16.52	16.31
**1986-07-27**	21.06	21.68	21.41	21.35
**1987-07-26**	14.95	15.39	15.78	15.34
**1988-07-24**	15.96	16.60	16.22	16.31
**1989-07-23**	25.96	25.59	24.42	25.03
**1990-07-22**	24.28	24.33	24.17	24.10
**1991-07-28**	20.78	21.08	21.00	20.77
**1992-07-26**	21.86	22.64	22.46	22.48
**1993-07-25**	15.72	15.73	15.52	15.28
**1994-07-24**	26.21	25.74	24.73	23.99
**1995-07-23**	18.07	18.51	18.82	18.84
**1996-07-21**	21.10	21.21	21.11	20.96
**1997-07-27**	20.44	20.28	19.94	19.97

**1999-07-25**	23.11	23.50	23.78	23.69
**2000-07-23**	21.62	21.93	22.14	22.01
**2001-07-29**	22.64	22.81	23.00	22.92
**2002-07-29**	26.77	26.80	26.72	26.30
**2003-07-27**	18.37	18.39	18.45	18.60
**2004-07-25**	18.33	18.97	19.09	18.97
**2005-07-24**	18.30	19.26	19.93	20.20
**2006-07-23**	23.40	23.42	23.33	23.14
**2007-07-29**	18.39	17.94	18.17	17.45
**2008-07-27**	23.45	23.63	23.94	23.29
**2009-07-26**	20.35	20.78	20.87	20.70
**2010-07-25**	17.85	18.38	18.65	18.36
**2011-07-24**	16.38	16.33	16.29	16.50
**2012-07-22**	18.42	18.53	18.72	18.68
**2013-07-21**	25.57	25.88	25.75	25.67
**2014-07-27**	23.20	23.32	23.58	22.60
**2015-07-26**	13.97	14.16	14.22	13.64
**2016-07-21**	22.15	22.09	21.33	20.73
**2017-07-23**	16.57	16.59	16.45	16.07
**2018-07-29**	21.14	21.45	21.50	21.48
**2019-07-28**	18.80	18.95	18.87	19.03
**2021-07-18**	22.92	23.18	23.34	23.23
**2022-07-24**	24.66	25.15	25.33	25.47
**2023-07-23**	19.31	20.02	20.09	20.21

Supplementary Table S2: WBGT values [°C] at 1500, 1600, 1700 and 1800 h local time on the dates of the Tour de France stage race through Nimes

Date	WBGT 1500h	WBGT 1600h	WBGT 1700h	WBGT 1800h
**1986-07-18**	23.35	23.56	22.73	22.13
**1986-07-19**	20.52	20.64	20.66	20.48
**2004-07-18**	22.22	21.45	21.05	20.71
**2008-07-18**	22.22	22.64	22.92	22.89
**2008-07-19**	23.85	23.84	23.37	22.73

**2014-07-20**	21.02	21.62	21.59	20.60
**2019-07-23**	27.94	27.73	27.50	26.84
**2021-07-08**	22.63	21.49	21.61	21.70
**2021-07-09**	23.01	23.45	23.54	23.44

Supplementary Table S3: WBGT values [°C] at 1500, 1600, 1700 and 1800 h local time on the dates of the Tour de France stage race through Col du Tourmalet

Date	WBGT 1500h	WBGT 1600h	WBGT 1700h	WBGT 1800h
**1974-07-16**	14.16	14.52	14.49	14.66
**1974-07-17**	11.02	11.20	10.69	10.83
**1975-07-08**	17.55	17.40	16.97	16.99
**1976-07-11**	18.58	19.19	18.80	18.74
**1977-07-02**	17.86	17.51	17.86	17.62
**1978-07-11**	16.04	15.82	16.04	15.92
**1980-07-10**	10.39	10.96	11.17	11.02
**1983-07-11**	22.27	23.59	24.17	24.10
**1985-07-16**	16.52	17.06	17.50	17.56
**1986-07-16**	19.49	20.29	20.74	20.58
**1988-07-18**	21.82	22.51	21.92	22.22
**1989-07-11**	16.36	16.72	16.70	16.63
**1990-07-17**	21.24	21.91	22.01	22.12
**1991-07-19**	21.61	21.50	21.48	21.38
**1993-07-22**	15.47	15.55	15.49	15.40
**1994-07-15**	22.42	22.47	22.62	22.43
**1995-07-18**	21.09	21.52	21.51	21.35
**1997-07-14**	19.69	20.20	20.42	19.91
**1998-07-21**	16.49	16.69	15.77	15.53
**1999-07-21**	19.84	20.76	19.99	19.40
**2001-07-22**	20.93	21.25	21.26	21.42
**2003-07-21**	20.10	20.00	20.02	19.94
**2006-07-13**	22.97	23.18	22.85	22.82
**2008-07-14**	15.27	16.07	16.12	15.95
**2009-07-12**	20.27	20.91	20.99	20.86
**2010-07-20**	20.75	22.06	21.99	21.34
**2010-07-22**	15.92	15.21	14.01	14.74
**2011-07-17**	13.05	12.78	12.99	12.79
**2012-07-18**	21.39	21.88	22.10	21.72

**2014-07-24**	21.00	21.76	21.26	20.77
**2015-07-15**	22.55	22.85	22.72	22.53
**2016-07-09**	22.06	22.53	22.55	22.70
**2018-07-27**	22.93	22.35	22.23	21.58
**2019-07-20**	22.25	22.20	21.49	21.14
**2021-07-15**	16.18	15.95	15.49	15.97
**2023-07-06**	20.87	20.90	18.65	18.21

Supplementary Table S4: WBGT values [°C] at 1500, 1600, 1700 and 1800 h local time on the dates of the Tour de France stage race through Toulouse

Date	WBGT 1500h	WBGT 1600h	WBGT 1700h	WBGT 1800h
**1978-07-12**	21.88	22.47	22.80	22.85
**1985-07-15**	20.95	21.13	22.05	22.06
**1985-07-16**	19.19	20.28	20.78	20.80
**1989-07-13**	22.59	22.46	22.73	22.74
**2003-07-17**	22.20	22.57	22.69	22.62
**2003-07-19**	27.48	27.78	27.79	27.88
**2008-07-12**	17.01	17.08	16.39	15.44
**2008-07-13**	16.67	16.73	16.75	16.41
**2019-07-17**	23.85	23.78	24.17	23.69
**2019-07-18**	20.57	21.28	21.52	21.41

Supplementary Table S5: WBGT values [°C] at 1500, 1600, 1700 and 1800 h local time on the dates of the Tour de France stage race through Bordeaux

Date	WBGT 1500h	WBGT 1600h	WBGT 1700h	WBGT 1800h
**1974-07-18**	17.77	17.82	17.99	17.01
**1975-07-04**	18.38	18.78	19.01	18.27
**1976-07-14**	23.25	24.35	24.06	23.74
**1977-07-05**	23.32	23.41	23.37	22.71
**1977-07-07**	19.16	19.37	20.50	20.38
**1978-07-06**	15.72	15.02	14.71	14.0
**1978-07-08**	17.78	18.36	19.25	18.76
**1979-07-01**	14.59	14.61	14.68	14.37
**1980-07-07**	20.31	20.60	20.34	18.76
**1981-07-02**	19.04	18.66	19.18	16.90
**1982-07-13**	23.89	24.66	23.98	21.59
**1983-07-09**	26.67	27.16	27.30	27.06
**1983-07-10**	28.36	28.70	29.05	28.44
**1984-07-07**	23.82	25.19	25.72	24.25
**1985-07-18**	21.06	21.08	20.97	20.04
**1986-07-14**	20.69	21.09	21.30	20.04
**1987-07-12**	25.15	25.56	25.55	25.26
**1988-07-19**	23.59	23.78	24.03	23.77
**1989-07-18**	23.55	23.72	23.11	22.82
**1990-07-19**	23.73	23.96	23.97	23.48
**1992-07-07**	19.45	19.45	19.38	18.74
**1993-07-23**	21.94	22.25	21.84	21.32
**1995-07-20**	28.74	29.21	29.07	27.43
**1996-07-19**	24.08	24.27	24.39	24.05
**1996-07-20**	23.81	23.91	24.10	23.45
**1997-07-12**	22.69	23.19	23.36	23.34
**1999-07-22**	18.00	18.37	18.35	17.67
**2003-07-24**	22.48	22.41	22.50	21.24
**2003-07-25**	23.94	23.64	23.74	22.57

**2006-07-11**	25.00	25.11	25.14	24.41
**2010-07-23**	19.11	19.25	19.04	18.81
**2010-07-24**	21.00	20.65	20.51	19.70
**2023-07-07**	26.75	26.97	27.14	24.37

Supplementary Table S6: WBGT values [°C] at 1500, 1600, 1700 and 1800 h local time on the dates of the Tour de France stage race through Alpe d'Huez

Date	WBGT 1500h	WBGT 1600h	WBGT 1700h	WBGT 1800h
**1976-07-04**	15.78	16.23	16.47	16.19
**1977-07-19**	15.18	16.03	15.88	15.47
**1978-07-16**	16.72	17.36	17.10	17.12
**1979-07-16**	15.62	15.40	14.35	14.21
**1979-07-17**	15.10	15.03	14.71	14.76
**1981-07-14**	14.49	15.26	15.22	15.03
**1982-07-20**	17.08	17.33	16.85	17.29
**1983-07-18**	18.05	18.21	18.36	18.22
**1984-07-16**	10.61	10.71	10.78	10.68
**1986-07-21**	14.99	15.51	15.21	15.15
**1987-07-21**	12.54	13.19	12.79	12.79
**1988-07-15**	9.39	9.72	9.18	9.20
**1989-07-19**	16.39	16.83	17.03	16.87
**1990-07-11**	14.52	15.34	15.62	15.34
**1991-07-23**	16.97	17.14	16.25	16.75
**1992-07-19**	20.05	20.68	20.75	20.70
**1994-07-19**	14.68	13.88	13.01	14.21
**1995-07-12**	17.07	17.30	18.01	17.97
**1997-07-19**	11.38	11.43	10.75	10.36
**1999-07-14**	13.97	14.25	13.92	13.91
**2001-07-17**	13.07	13.46	13.58	13.05
**2003-07-13**	19.56	19.87	19.74	19.50
**2004-07-21**	17.78	17.73	17.80	17.76

**2006-07-18**	18.91	19.48	17.58	17.84
**2008-07-23**	15.22	15.62	15.48	15.29
**2011-07-22**	12.98	13.22	12.80	12.15
**2013-07-08**	17.93	18.46	18.10	16.93
**2013-07-18**	15.71	15.82	14.79	14.31
**2015-07-25**	14.56	14.10	13.89	13.40
**2018-07-19**	17.99	17.98	18.21	17.88
**2022-07-14**	19.54	19.82	19.77	19.54
